# Secondary Metabolites from Fungi *Microsphaeropsis* spp.: Chemistry and Bioactivities

**DOI:** 10.3390/jof9111093

**Published:** 2023-11-09

**Authors:** Guodong Song, Zhibin Zhang, Xuenan Niu, Du Zhu

**Affiliations:** 1 Key Laboratory of Protection and Utilization of Subtropical Plant Resources of Jiangxi Province, College of Life Science, Jiangxi Normal University, Nanchang 330022, China; songgd2079@163.com (G.S.); 15832057270@163.com (X.N.); 2 Key Laboratory of Bioprocess Engineering of Jiangxi Province, College of Life Sciences, Jiangxi Science and Technology Normal University, Nanchang 330013, China

**Keywords:** *Microsphaeropsis*, secondary metabolites, bioactivities

## Abstract

*Microsphaeropsis*, taxonomically classified within the kingdom fungi, phylum Ascomycota, subphylum Deuteromycotina, class Coelomycetes, order Sphaeropsidales, and family Sphaeropsidaceae, exhibit a ubiquitous distribution across various geographical regions. These fungi are known for their production of secondary metabolites, characterized by both structural novelty and potent biological activity. Consequently, they represent a significant reservoir for the advancement of novel pharmaceuticals. In this paper, a systematic review was present, marking the analysis of secondary metabolites synthesized by *Microsphaeropsis* reported between 1980 and 2023. A total of 112 compounds, comprising polyketones, macrolides, terpenoids, and nitrogen-containing compounds, were reported from *Microsphaeropsis*. Remarkably, among these compounds, 49 are novel discoveries, marking a significant contribution to the field. A concise summary of their diverse biological activities was provided, including antibacterial, antitumor, and antiviral properties and other bioactivities. This analysis stands as a valuable reference, poised to guide further investigations into the active natural products derived from *Microsphaeropsis* and their potential contributions to the development of medicinal resources.

## 1. Introduction

*Microsphaeropsis*, taxonomically classified within the phylum Ascomycetes, subphylum Deuteromycotina, class Coelenterata, order Sphaeropsidales, and family Sphaeropsidaceae in the fungal taxonomy [1], are common plant pathogens widely distributed in nature [2]. For a long time, the genus *Microsphaeropsis* was not accurately recognized, leading to the misplacement of many fungi within the genus *Coniothyrium*. In 1980, Sutton meticulously elucidated the characteristics of the *Microsphaeropsis* genus. He subsequently reclassified species previously assigned to the genus *Coniothyrium* under *Microsphaeropsis* and renamed the genus [3,4]. Subsequently, additional members of the genus *Microsphaeropsis* have been unearthed and taxonomically elucidated. As of now, the repository of the Species Fungorum database (https://speciesfungorum.org/Names/Names.asp (accessed on 2 November 2023)) registers a total of 54 species within the genus *Microsphaeropsis*. It is evident that fungi within the genus *Microsphaeropsis* exhibit remarkable biodiversity, with a high likelihood of containing structurally novel and biologically active secondary metabolites.

Fungi have been recognized for their remarkable capacity to synthesize secondary metabolites, and the extraction of natural products from fungal secondary metabolites offers significant advantages in terms of yield, economic viability, and environmental sustainability [5,6]. Following the taxonomic redefinition of the *Microsphaeropsis* genus by Sutton in 1980, various structural categories of compounds, including polyketides, terpenoids, nitrogen-containing compounds, macrolides, and others, were isolated from 2 known and 17 unknown species of *Microsphaeropsis*, and these demonstrated excellent antibacterial, antifungal, cytotoxic, and other activities (Table 1). This repertoire encompasses numerous structurally novel compounds. However, there exists a current paucity of both domestic and international research focused on the secondary metabolites originating from this genus of fungi. This thesis presents the review of the spectrum of secondary metabolites derived from fungi within the *Microsphaeropsis* genus, shedding light on their structural diversity and biological activities. This effort not only advances our understanding of these fungal secondary metabolites but also lays the foundation for future exploration and exploitation of the resources within this genus.

## 2. Types of Chemical Structures

### 2.1. Polyketones

**Figure 1:**
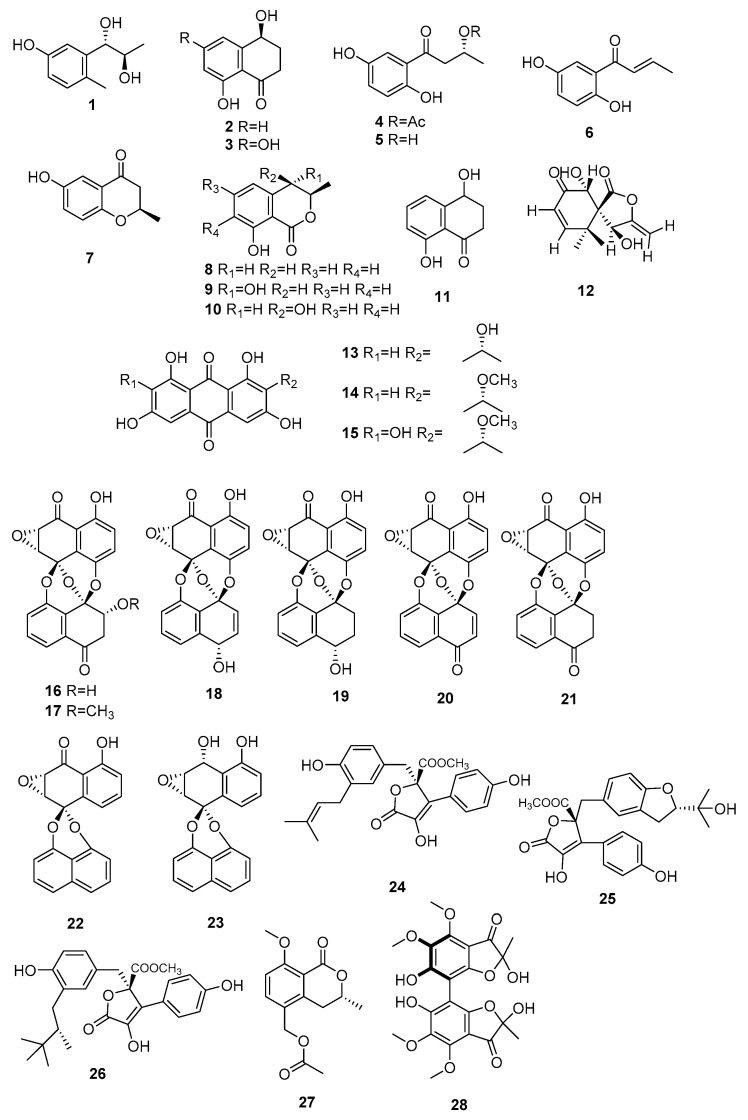

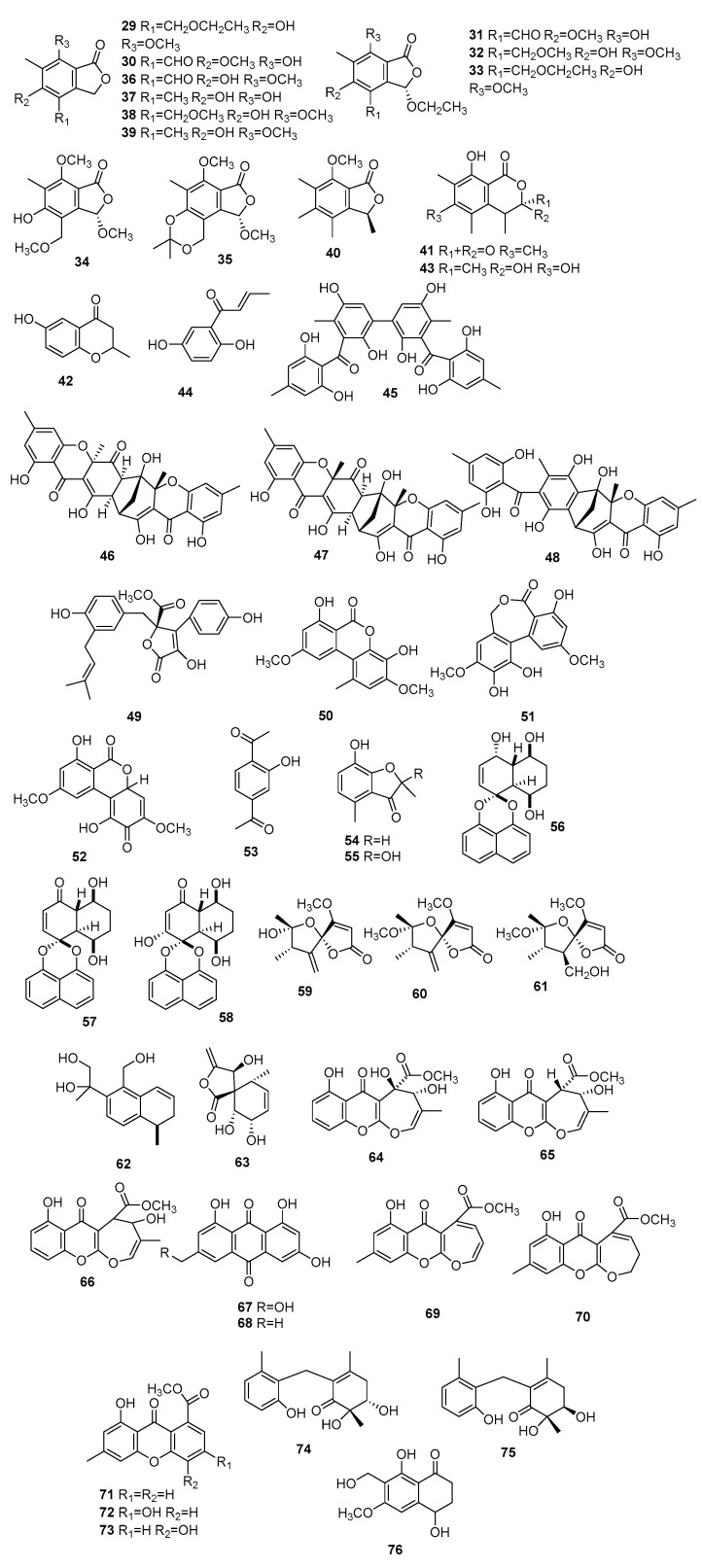
Chemical structures of polyketone compounds **1**–**76**.

Polyketones represent a class of secondary metabolites characterized by their exceptionally diverse structures. These compounds are generated through a sequence of Claisen condensation reactions involving short-chain acyl-CoA molecules such as acetyl-CoA and malonyl-CoA. Their distinctive activities have positioned them as a prominent source for both the treatment of human diseases and the development of novel pharmaceuticals. Seventy-six polyketone compounds were found in *Microsphaeropsis* (Figure 1), encompassing pyranones, furanones, naphthoquinones, anthraquinones, phenylpropanoids, and coumarins. In terms of sheer chemical diversity, polyketones emerge as the most extensively documented natural products among the fungi within the *Microsphaeropsis* genus.

### 2.2. Terpenoids

**Figure 2:**
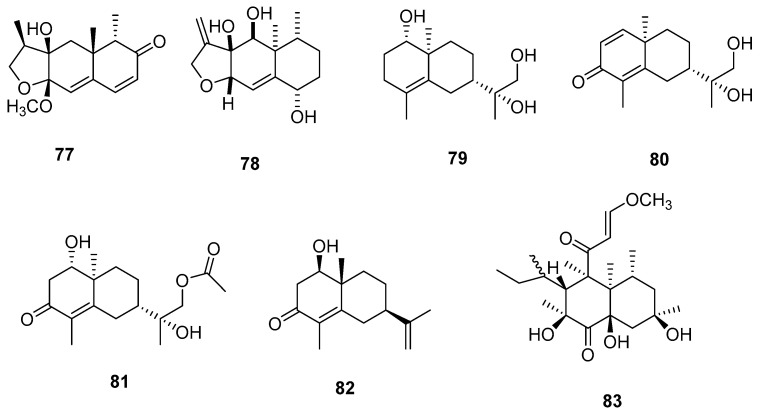
Chemical structures of terpenoid compounds **77**–**83**.

Terpenoids represent a class of natural compounds characterized by their foundational isoprene or isopentane unit structures. They exhibit unparalleled structural diversity and comprise the most extensive group of natural compounds, offering a wide array of activities, including anti-inflammatory, antibacterial, and antitumor properties [32,33]. Seven sesquiterpenes were discovered within *Microsphaeropsis* (Figure 2), and this collection includes eucalyptus sesquiterpenes, airimo phenolic sesquiterpenes, and sesterterpene.

### 2.3. Macrolide

**Figure 3:**
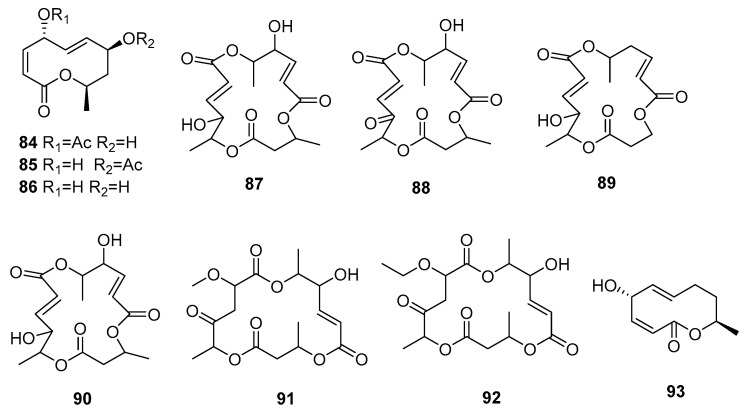
Chemical structures of macrolide compounds **84**–**93**.

Macrolide compounds represent a class of multi-carbon compounds distinguished by the presence of a lactone ring within their molecular structure. They are notable for their pronounced anti-inflammatory, anti-tumor, and antibacterial activities [34]. A comprehensive review of 10 macrolides (Figure 3), comprising two distinct structural types, 10-member macrolides and 16-member macrolides, is provided. These are the first natural-product-bearing three lactone groups in the molecule among the 16-membered macrocyclic antibiotics, showcasing remarkable anti-leukocyte activity.

### 2.4. Nitrogen Compounds

**Figure 4:**
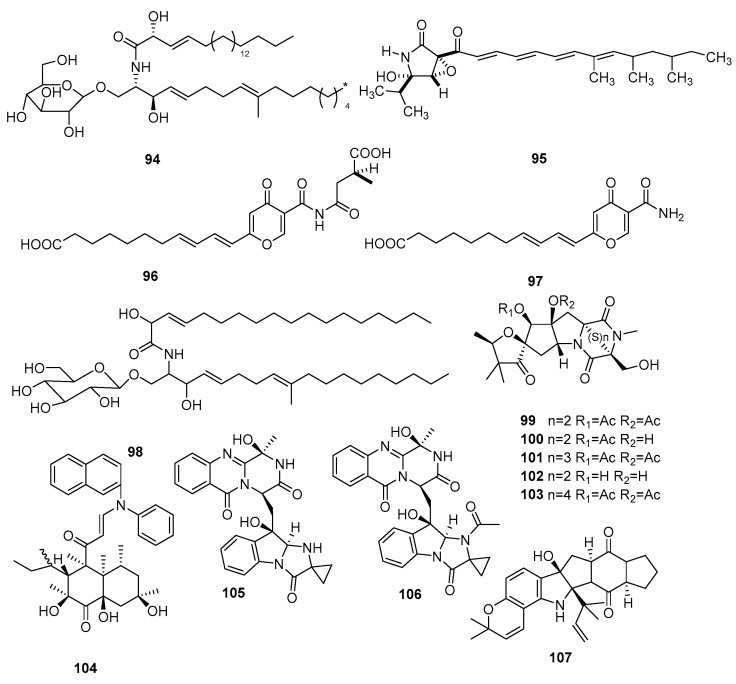
Chemical structures of nitrogen compounds **94**–**107**.

Nitrogen-containing compounds represent another prevalent and exceptionally diverse class of secondary metabolites. They are distinguished by their intricate cyclic structures and demonstrate substantial biological activities against bacteria, fungi, and tumor cells [35]. This study offers an extensive review of 14 nitrogen-containing compounds, encompassing a range of subtypes (Figure 4), including organic amines alkaloids, pyrrole alkaloids, pyrazine alkaloids, imidazolone alkaloids, indole alkaloids.

### 2.5. Other Classes

**Figure 5:**
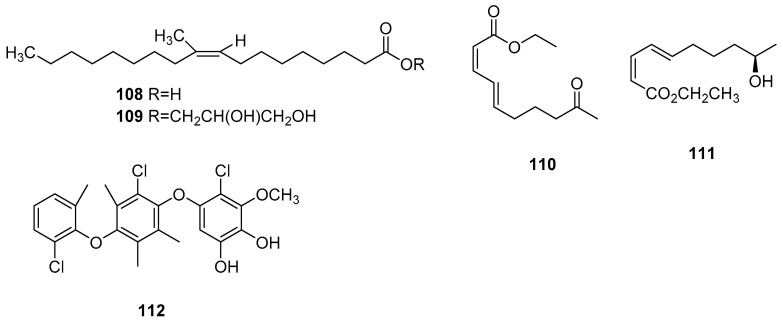
Chemical structures of compounds **108**–**112** of other classes.

In addition to their primary secondary metabolites, *Microsphaeropsis* fungi also generate a limited quantity of fatty acids and chlorine-containing compounds (Figure 5). Among these, compound 112 has demonstrated noteworthy antibacterial activity.

## 3. Biological Activities

Numerous studies have revealed the isolation of a plethora of bioactive compounds from the secondary metabolites of *Microsphaeropsis* fungi [7,9]. These compounds encompass a wide spectrum of activities, notably including antifungal, antibacterial, cytotoxic, cell adhesion inhibition, antiviral, antimalarial, and antioxidant activities [15,18,31]. Furthermore, some of these compounds exhibit a substantial potential for their development into novel pharmaceuticals.

### 3.1. Antifungal Activity

Qin et al. (2017) successfully isolated four compounds from the endophyte fungus *Microsphaeropsis arundinis* PH 30472, which inhabits the plant *Panax notoginseng*. These compounds comprise a novel isochroman derivative, erythro-2-methyl-5-hydroxy -phenylpropane-7,8-diol (**1**) and three known compounds: (4*S*)-4,8-dihydroxy -3,4-dihydro-1(2*H*)-naphthalen-1-one (**2**), (4*S*)-4,6,8-trihydroxy-3,4-dihydro-1(2*H*)- naphthalen-1-one (**3**), and chrysogeside D (**94**). Notably, compounds **3** and **94** were discovered for the first time in *Microsphaeropsis*. The research team conducted evaluations of the antifungal activity of these compounds. Compound **1** and compound **94** exhibited moderate antifungal activity against *A. tenuissima* with minimum inhibitory concentrations (MICs) at 64 μg/mL [7].

The novel compound microsphaeropsisin (**77**), along with the known compounds (*R*)-mellein (**8**), (3*R*,4*S*)-hydroxymellein (**9**), (3*R*,4*R*)-hydroxymellein (**10**), and 4,8-dihydroxy-3,4-dihydro-2*H*-naphthalen-1-one (**11**), were successfully isolated from the fungus *Microsphaeropsis* sp. H5-50, which inhabits the marine sponges *M. incrustans*. Notably, both compounds displayed significant antifungal activity against *E. repens* and *U. violacea* in agar diffusion assays [9].

Sommart et al. (2012) isolated nineteen compounds from the endophyte fungus *M. arundinis* PSU-G18, found within the plant *Garcinia hombroniana* [15]. Among these compounds, one new modiolin, microsphaerodiolin (**110**), and seven new phthalides, microsphaerophthalides A-G (**29**–**35**), were identified, alongside eleven known compounds (**36**–**44**, **93**, and **111**). Compound **44** exhibited remarkable antifungal activity, with an IC_50_ value of 8 μg/mL. Additionally, microsphaerophthalides A (**29**) and microsphaerophthalides E (**33**) displayed moderate antifungal activity against *M. gypseum SH-MU-4* and *C. neoformans*, both with MIC values of 64 μg/mL. Decaspirone (**57**), isolated from the endophyte fungus *Microsphaeropsis* sp. 7291, demonstrated significant antifungal activity against *M. violaceum*, evidenced by inhibition zone diameters measuring 18 mm [18].

A novel polychlorinated triphenyl diether, microsphaerol (**112**), was successfully isolated from the endophyte fungus *Microsphaeropsis* sp. 7820, found inhabiting the plant *Salsola oppositifolia*. Remarkably, compound **112** exhibited moderate antifungal activity against *M. violaceum*, resulting in inhibition zone diameters measuring 9 mm [31].

Liu et al. (2020) isolated two novel metabolites, microketides A (**74**) and microketides B (**75**), from the fungus *Microsphaeropsis* sp. RA10-14, and both compounds exhibited significant antifungal activity against *Candida albicans*, *Colletotrichum truncatum*, *Gloeosporium musarum*, and *Pestalotia calabae* [21]. Microketides A (**74**) exhibited IC_50_ values of 1.56 μg/mL, 1.56 μg/mL, 3.13 μg/mL, and 1.56 μg/mL, respectively. Microketides B (**75**) displayed equal IC_50_ values of 3.13 μg/mL.

### 3.2. Antibacterial Activity

Four metabolites, namely microsphaerins A–D (**45**–**48**), were isolated from *Microsphaeropsis* sp. F2076 and *Microsphaeropsis* sp. F2078. Notably, the four microsphaerins, A, B, C, and D, exhibited significant antibacterial activity against Methicillin-resistant *Staphylococcus aureus* (MRSA), with IC_50_ values of 3 μM, 3 μM, 5 μM, and 1 μM, respectively. To delve deeper into their antibacterial activity, microsphaerins D (**48**) was selected for further investigation, encompassing Gram-positive and Gram-negative bacteria. Results revealed that it demonstrated moderate antibacterial activity against various Gram-positive bacteria; however, it displayed inactivity against Gram-negative bacteria, except for *K. pneumoniae* [16].

Five new metabolites, namely palmarumycin M_1_ (**56**), palmarumycin M_2_ (**58**), papyracillic acid C (**61**), microsphaeropsins A (**78**), and microsphaeropsins B (**62**), were successfully isolated, alongside three known compounds, decaspirone (**57**), papyracillic acids A (**59**), and papyracillic acids B (**60**), from the endophyte fungus *Microsphaeropsis* sp. 7291, which resides within the plant *Pilgerodendron uviferum*. Of significance, compounds **56**, **57**, and **58** exhibited moderate antibacterial activity against *B. megaterium*, resulting in inhibition zone diameters of 6 mm, 6 mm, and 7 mm, respectively [18].

Krohn et al. (2009) conducted a comprehensive study resulting in the isolation of several metabolites from the endophyte fungus *Microsphaeropsis* sp. 8875 residing in the plant *Lycium intricatum* [20], and they isolated three new compounds, microsphaeropsones A-C (**64**–**66**), in addition to two known compounds, citreorosein (**67**) and emodin (**68**). Moreover, from a *Microsphaeropsis* sp. 7177 strain sourced from the plant *Zygophyllum fortanesii*, they identified two new metabolites, 3,4-dihydrofusidienol A (**70**) and microsxanthone (**73**), along with three known compounds, fusidienol A (**69**), 8-hydroxy-6-methyl-9-oxo-9*H*-xanthene-1-carboxylic acid methyl ester (**71**), and methyl 3,8-dihydroxy-6-methyl-9-oxo-9*H*-xanthene-1-carboxylate (**72**). Remarkably, all these compounds exhibited significant antibacterial activity, particularly against the Gram-negative bacterium *E. coli*. Microsphaerol (**112**), derived from the endophyte fungus *Microsphaeropsis* sp. 7820, displayed moderate antibacterial activity against *B. megaterium* and *E. coli*, evidenced by inhibition zone diameters measuring 9 mm and 8 mm, respectively [31]. 

Two novel metabolites, microketide A (**74**) and microketide B (**75**), were successfully isolated from the fungus *Microsphaeropsis* sp. RA10-14, which inhabits the gorgonian *Anthogorgia ochracea* from the South China Sea. Both compounds exhibited significant antibacterial and antifungal activities, with a notable emphasis on their antibacterial effects. Specifically, compound **74** demonstrated significant antibacterial activity against *B. subtilis*, *B. megaterium*, *E. aerogenes*, *K. rhizophila*, and *P. aeruginosa*, all with equal MIC values of 0.19 μg/mL, mirroring the potency of ciprofloxacin [21]. Ciprofloxacin is used for the treatment of a wide range of infections and has been shown to be active against various Gram-positive and Gram-negative bacteria [36,37,38]. 

The new isomer, 3′-O-demethylpreussomerin I (**16**), was isolated alongside seven known compounds from the fungus *Microsphaeropsis* sp. BCC 3050. This fungal strain was collected from the lichen *Dirinaria applanata*. The known compounds in this collection include preussomerins E (**18**), F (**19**), G (**20**), H (**21**), I (**17**), deoxypreussomerins A (**22**), and bipendensin (**23**). Remarkably, all compounds, except compound **23**, exhibited activities against *M. tuberculosis*, and compounds **16**, **17**, and **18** showed moderate antibacterial activity, with IC_50_ values of 25 μg/mL, 12.5 μg/mL, and 25 μg/mL, respectively. Compounds **19**, **20**, **21,** and **22** showed significant antibacterial activity, with IC_50_ values of 3.12 μg/mL, 3.12 μg/mL, 6.25 μg/mL, and 1.56 μg/mL, respectively [12]. Additionally, chrysogeside D (**94**), sourced from the endophyte fungus *Microsphaeropsis arundinis* PH 30472, showed moderate antibacterial activity against *E. coli* [7].

Three novel ent-eudesmane sesquiterpenoids, namely arundinols A (**79**), B (**80**), and C (**81**), were isolated alongside three known compounds from the endophyte fungus *M. arundinis* E12-2112, which resides within the plant *Ulmus macrocarpa*. Among the known compounds, one isochroman-1-one, arundinone A (**27**), a polyoxygenated benzofuran-3(2*H*)-one dimer, arundinone B (**28**), and 1*β*-hydroxy-*α*-cyperone (**82**) were identified. Significantly, compound **82** displayed moderate antibacterial activity against *S. aureus* with an value of 11.4 μg/mL [14].

### 3.3. Cytotoxic Activity

A new metabolite, (*R*)-1′-(2,5-dihydroxyphenyl)-1′-oxobutan-3′-ylacetate (**4**), was isolated, alongside six known compounds, (*R*)-1-(2,5-dihydroxyphenyl)-3-hydroxybut -anone (**5**), 1-(2,5-dihydroxyphenyl)-2-buten-1-one (**6**), (*R*)-6-hydroxy-2-methyl-4-chrom -anone (**7**), modiolide D (**84**), modiolide E (**85**), and modiolide A (**86**). These compounds were obtained from the endophyte fungus *M. arundinis*, residing within the plant *Paepalanthus planifolius* [8,23]. Additionally, Botero et al. (2020) reported, for the first time, that compound **5** exhibited moderate cytotoxic activity against murine breast adenocarcinoma (LM3), with IC_50_ values of 36.83 ± 4.86 μg/mL, while compound **6** displayed moderate cytotoxic activity against human breast cancer (MCF-7), with IC_50_ values of 33.95 ± 3.62 μg/mL [8].

Keusgen et al. (1996) successfully isolated a cerebroside, namely *N*-2”-hydroxy- 3’*E*-octadecenoyl-1-*O*-*β*-*D*-glucopyranosyl-9-methyl-4*E*,8*E*-sphingadiene (**98**), through alterations of the culture medium for the fungus *M. olivacea* F010 [39]. Remarkably, at a concentration of 2 μg/mL, compound **98** demonstrated significant cytotoxic activity against murine leukemic cells (L1210), achieving an inhibition ratio of 90% [28].

Three novel metabolites, TAN-1496 A (**99**), C (**101**), and E (**103**), in conjunction with two known compounds, TAN-1496 B (**100**) and D (**102**), were successfully isolated from the fungus *Microsphaeropsis* sp. FL-16144. Extensive research has revealed that these compounds act as specific inhibitors of calf thymus Topo I [40]. Notably, even at high concentrations, these compounds did not inhibit the function of Topo II. They exhibited a significant inhibition of the growth of various murine and human tumor cells, including P815 murine mastcytoma, EL4 murine lymphoma, Bl6 murine melanoma, WiDr human colon adenocarcinoma, and A549 human lung carcinoma [29]. Additionally, these compounds displayed activity against Gram-positive bacteria [29].

It has been widely reported that cell adhesion molecules play pivotal roles in numerous physiological and biochemical processes, ranging from cancer [41,42,43] to inflammation [44]. In a remarkable discovery, Hayashi et al. (1995) isolated two new 16-membered macrolides, namely macrosphelide A (**87**) and macrosphelide B (**88**), from the fungus *Microsphaeropsis* sp. FO-5050 [24]. To explore the impact of macrosphelide A and B on cell adhesion, experiments were conducted using the dose-dependently inhibited adhesion of HL-60 cells to human umbilical vein endothelial cells (HUVECs) stimulated with LPS. These compounds exhibited significant cytotoxicity, with IC_50_ values of 3.5 μM and 36 μM, respectively. Notably, in vitro cell growth assays showed that these compounds had no discernible effect on the growth of cells, including P388 leukemia, human prostate tumor cells, and L929 fibroblast cells, even when administered at a high dose of 200 mg/kg for 5 days [45]. Takamatsu’s (1997) group isolated two more 16-membered macrolide metabolites, macrosphelide C (**89**) and macrosphelide D (**90**) from the fungus *Microsphaeropsis* sp. FO-5050 [25]. Macrosphelide C (**89**) and macrosphelide D (**90**) exhibited moderate cytotoxic activity, with IC_50_ values of 67.5 μM and 25 μM, respectively. Fukami et al. (1998) continued their exploration of the fungus *Microsphaeropsis* sp. FO-5050 and made further discoveries [10]. They isolated three new metabolites, macrosphelide J (**91**), macrosphelide K (**92**), and 6-epi-5’-hydroxymycosporulone (**12**). Although these compounds displayed cytotoxic activity, their IC_50_ values exceeded 100 μg/mL. Collectively, these findings underscore the exceptional potential of the fungus *Microsphaeropsis* sp. FO-5050 as a source of novel macrolides with cytotoxic properties. These substances hold high promise for future development in the treatment of leukemia.

Singh et al. (1994) previously reported that compounds **18**, **19**, and **20** possess the capability to inhibit Ras farnesyl-protein transferase [46]. Seephonkai et al. (2002) corroborated this finding in their research [12]. Furthermore, compounds **16**–**21** have demonstrated significant cytotoxicity against a spectrum of cell lines, including human epidermoid carcinoma (KB cells), human breast cancer (BC-1 cells), and African green monkey kidney fibroblast (vero cells). Compound **28**, sourced from the endophyte fungus *M. arundinis* E12-2112, has exhibited moderate cytotoxicity against human bladder carcinoma cells (T24) and human lung carcinoma cells (A549), characterized by IC_50_ values of 35.4 μg/mL and 81.6 μg/mL, respectively [14].

### 3.4. Other Biological Activities

Seven known compounds, namely butyrolactone I (**49**), graphislactone A (**50**), ulocladol (**51**), botrallin (**52**), 2,5-diacetylphenol (**53**), 7-hydroxy-2,4-dimethyl-3(2*H*)- benzofuranone (**54**), and enalin (**55**), were isolated from the endophyte fungus *M. olivacea*, which resides within the plant *Pilgerodendron uviferum*. Remarkably, graphislactone A (**50**) and botrallin (**52**) exhibited significant activity against acetylcholinesterase (AChE), characterized by IC_50_ values of 8.1 μg/mL and 6.1 μg/mL, respectively. However, all these compounds displayed no discernible antibacterial or antifungal activities [17].

Fukami et al. (2000) isolated a novel anti-influenza virus antiviral, known as 10-norparvulenone (**76**), from the fungi *Microsphaeropsis* sp. FO-5050 [22]. Interestingly, this compound did not exhibit any discernible antibacterial activity. However, experimental results revealed its remarkable ability to inhibit the replication of the influenza virus A/PR/8/34 strain within Madin-Darby Canine Kidney (MDCK) cells. The outcomes of these experiments were striking: In the absence of the compound, only 27.2% of the cells survived. In contrast, when the compound was introduced at a concentration of 25 μg/mL, cell survival significantly increased to 64.8%. This groundbreaking discovery suggests that compound **76** holds great promise as a novel class of anti-influenza virus drugs, representing a potential breakthrough in the realm of influenza treatment.

Bradykinin, an endogenous peptide, exerts its effects through specific cell receptors, giving rise to a plethora of physiological reactions, including pain, allergic responses, and muscle contractions. Bradykinin antagonists hold the potential to inhibit these physiological reactions, offering prospects for treating conditions such as inflammatory edema, rhinitis, and asthma. In a significant discovery, a novel non-peptide bradykinin-binding inhibitor, L-755,807 (**95**), was isolated from the endophyte fungus *Microsphaeropsis* sp. MF6057, found within the plant *Prosopis glandulosa*. Compound **95** demonstrated an IC_50_ value of 71 μM in binding to a cloned human B2 receptor with ^3^H-bradykinin, marking a noteworthy development in the pursuit of potential treatments [26].

Two novel *γ*-pyrone derivatives, microsphaerones A (**96**) and microsphaerones B (**97**), were successfully isolated from the fungus *Microsphaeropsis* sp. KMPB W-22. Interestingly, neither of these compounds exhibited significant cytotoxicity. Additionally, they displayed either no activity or only moderate activity against *S. littoralis* and *A. salina* [27].

Two novel betaenone derivatives (compounds **83** and **104**) and three fresh 1,3,6,8-tetrahydroxyanthraquinone congeners (**13**–**15**) were successfully extracted from the fungus *Microsphaeropsis* sp. KMPB W-22. Remarkably, all these compounds, with the exception of compound **104**, exhibited moderate inhibitory activity against protein kinases such as PKC, CDK4, and EGF-R, encompassing an IC_50_ range of 18.5–54.0 μM [11].

From a global vantage point, malaria continues to rank among the foremost causes of human mortality. Consequently, the current paramount emphasis remains on research and the development of targeted pharmaceutical interventions. Seephonkai et al. (2002) first reported on the in vitro activity of preussomerins against *Plasmodium falciparum* [12]. Compound **16**–**21** showed significant antiplasmodial activity, with an IC_50_ range of 0.32–3.44 μg/mL.

Compound **44**, sourced from the endophyte fungus *M. arundinis* PSU-G18, has exhibited significant antiplasmodial activity, boasting an IC_50_ value of 9.63 μg/mL. Notably, it also demonstrates significant radical scavenging capabilities, with an IC_50_ value of 0.018 mg/mL [15]. In another remarkable finding, palmarumycin M_1_ (**56**), decaspirone (**57**), and papyracillic acids A (**59**), all originating from the endophyte fungus *Microsphaeropsis* sp. 7291, have demonstrated anti-algal activity against *C. fusca*. Their inhibitory effects are reflected in inhibition zone diameters of 6 mm, 13 mm, and 7 mm, respectively [18]. Similarly, compound **112**, derived from the endophyte fungus *Microsphaeropsis* sp. 7820, has displayed anti-algal activity against *C. fusca*, characterized by an inhibition zone diameter of 8.5 mm [31]. Additionally, compounds **64**–**73**, apart from 8-hydroxy-6-methyl-9-oxo-9*H*-xanthene-1-carboxylic acid methyl ester (**71**), have demonstrated anti-algal activity against *C. fusca* [20]. Finally, microketides A (**74**) and microketides B (**75**), isolated from *Microsphaeropsis* sp. RA10-14, have exhibited moderate antiphytoplankton activity against *P. helgolandica*, showcasing IC_50_ values of 12.3 μg/mL and 16.8 μg/mL, respectively [21].

Furthermore, several compounds were isolated from fungi within the *Microsphaeropsis* genus, and their activities have yet to be determined. For instance, Liu et al. (2018) isolated six known metabolites, including butyrolactones I (**24**), butyrolactones IV (**25**), aspernolide D (**26**), fumiquinazolines L (**105**), fumiquinazolines N (**106**), and notoamide D (**107**), from the fungus *Microsphaeropsis* sp. CF09-11, sourced from marine sediment [13]. It is noteworthy that these compounds (**24**–**26** and **105**–**107**) were obtained from *Microsphaeropsis* for the first time. In a unique discovery, uncommon methyl-branched unsaturated fatty acids, 10-methyl-9*Z*-octadecenoic acid (**108**) and glyceride (**109**), were isolated from the endophyte fungus *M. olivacea*, which inhabits the marine sponge *Agelus sp*. This marks the first report of fungal fatty acids with methyl branches on a *cis*-double bond [30]. Additionally, three known metabolites, namely massarigenin A (**63**), papyracillic acids A (**59**), and papyracillic acids B (**60**), were isolated from the endophyte fungus *Microsphaeropsis* sp., originating from the plant *Arbutus unedo*. Importantly, this research led to the establishment of the absolute configuration of massarigenin A (**63**) [19].

## 4. Conclusions

**Figure 6:**
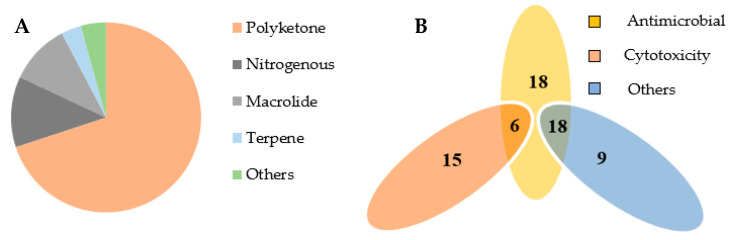
Structural classification (**A**) and activity classification (**B**) of secondary metabolites of *Microsphaeropsis* spp.

Since Sutton reclassified the genus *Microsphaeropsis* in 1980, fifty-four different species have been included. Currently, this genus has yielded an impressive array of secondary metabolites, with 112 secondary metabolites reported, of which 49 (41.96%) were identified for the first time. These compounds are distributed across various structural classes, including 76 polyketones (67.86%), 14 nitrogenous compounds (12.5%), 10 macrolides (8.9%), 7 terpenoids (6.25%), and 5 other compounds (4.46%), indicating polyketones are the predominant structural type (Figure 6A). Moreover, a significant proportion (57.89%) of these compounds exhibit notable biological activities, of which 42 compounds (60.6%) demonstrate both antibacterial and antifungal properties, while 21 (37.8%) compounds exhibit antitumor activities. Furthermore, several compounds display distinct activities, including scavenging oxygen-free radicals, algae removal, resistance to aging, and acetylcholinesterase inhibition (Figure 6B). It is important to highlight that many compounds exhibit multiple activities. For instance, compound **44** demonstrates antibacterial, antitumor, and certain oxygen-free radical scavenging activities.

Fungi occupy an exceptionally pivotal role in drug discovery [47]. Many renowned drug molecules, including penicillin and lovastatin, have originated from fungal sources [48]. However, the challenge of unearthing novel compounds of exceptional activity and expanding the repertoire of known compound activities remains unresolved. Given the abundance and significant innovation rate (41.96%) of secondary metabolites, *Microsphaeropsis* can be used as a potential repository for exploiting secondary metabolites. Accordingly, the following recommendations were proposed: (i) Employ non-directional activation strategies to activate the biosynthesis of secondary metabolites, such as the OSMAC (one strain many compounds) strategy [49], co-culture strategy [50], and epigenetic regulation strategy [51]. (ii) Harness targeted activation strategies to activate numerous dormant gene clusters within fungal genomes, such as target sequence promoter replacements [52], transcription regulatory factor knockouts [53], the heterologous expression of biosynthetic gene clusters [54], DNA-assembly technology [55], and ribosome engineering [56]. However, the genomic information of *Microsphaeropsis* has not been reported in the literature so far. Thus, whole gene sequencing and analysis will be the focus of further work. (iii) Expand the scope of activity screening of existing compounds and delve into their mechanism of action to verify their potential for pharmaceutical applications. For instance, microbetides A (**74**), a novel polyketone compound, demonstrates remarkable antibacterial activity against Gram-positive bacteria and Gram-negative bacteria, with an IC_50_ of 0.19 μg/mL, comparable to ciprofloxacin. Polyketone 10-norparvulone (**76**) exhibits the potential to effectively inhibit virus replications, positioning it as a promising candidate for a new anti-influenza drug. Macrolide compounds like macrosphelide A (**87**) and macrosphelide B (**88**), characterized by their unique chemical structures and cytotoxicity against leukemia, hold promise as novel anticancer drugs.

## Figures and Tables

**Table 1:** Summary of secondary metabolites of *Microsphaeropsis* spp.

No.	Chemical Name	Source	Bioactivities/Inactivity	Ref.
**1**	Erythro-2-methyl-5-hydroxyphenylpropane-7,8-diol	*M. arundinis* PH 30472	Antifungal activity against *Alternaria. tenuissima*	[7]
**2**	(4S)-4,8-dihydroxy-3,4-dihydro-1(2H)-naphthaln-1-one		Inactive	
**3**	(4S)-4,6,8-trihydroxy-3,4-dihydro-1(2H)-naphthalen-1-one		Inactive	
**4**	(*R*)-1′-(2,5-dihydroxyphenyl)-1′-oxobutan-3′-ylacetate	*M. arundinis*	Inactive	[8]
**5**	(*R*)-1-(2,5-dihydroxyphenyl)-3-hydroxybutanone		Anti-inflammatory activity and cytotoxicity activity against LM3	
**6**	1-(2,5-dihydroxyphenyl)-2-buten-1-one		Anti-inflammatory activity and cytotoxicity activity against LM3	
**7**	(R)-6-hydroxy-2-methyl-4-chromanone		Inactive	
**8**	(*R*)-mellein	*Microsphaeropsis* sp. H5-50	Antifungal activity against *Eurotium repens*	[9]
**9**	(3*R*,4*S*)-hydroxymellein		Antifungal activity against *Ustilago violacea*	
**10**	(3*R*,4*R*)-hydroxymellein		Antifungal activity against *E. repens* and *U. violacea*	
**11**	4,8-dihydroxy-3,4-dihydro-2H-naphthalen-1-one		Antifungal activity against *E. repens* and *U. violacea*	
**12**	6-ep-i 5′-hydroxymycosporulone	*Microsphaeropsis* sp. FO-5050	Cytotoxicity activity against HL-60	[10]
**13–15**	1,3,6,8-tetrahydroxyanthraquinone congenners	*Microsphaeropsis* sp. KMPB W-22	Inhibited protein kinases activity (PKC, CDK 4, and EGF-R)	[11]
**16**	3′-*O*-demethylpreussomerin I	*Microsphaeropsis* sp. BCC 3050	Antibacterial activity and antiplasmodial activity against *plasmodium falciparum* and cytotoxicity activity against KB, BC-1, and vero cells	[12]
**17**	Preussomerin I		-	
**18**	Preussomerin E		-	
**19**	Preussomerin F		-	
**20**	Preussomerin G		-	
**21**	Preussomerin H		-	
**22**	Deoxypreussomerin A		Antibacterial activity and antiplasmodial activity against *p. falciparum*	
**23**	Bipendensin		Inactive	
**24**	Butyrolactones I	*Microsphaeropsis* sp. CF09-11	Inactive	[13]
**25**	Butyrolactones IV		Inactive	
**26**	Aspernolide D		Inactive	
**27**	Arundinone A	*M. arundinis* E12-2112	Inactive	[14]
**28**	Arundinone B		Cytotoxicity activity against T24 and A549	
**29**	Microsphaerophthalides A	*M. arundinis* PSU-G18	Antifungal activity against *Microsporum gypseum* SH-MU-4, and *Cryptococcus neoformans*	[15]
**30**	Microsphaerophthalides B		Inactive	
**31**	Microsphaerophthalides C		Inactive	
**32**	Microsphaerophthalides D		Inactive	
**33**	Microsphaerophthalides E		Antifungal activity against *Microsporum gypseum* SH-MU-4 and *Cryptococcus neoformans*	
**34**	Microsphaerophthalides F		Inactive	
**35**	Microsphaerophthalides G		Inactive	
**36**	Deoxycyclopaldic acid		Inactive	
**37**	5,7-dihydroxy-4,6- dimethyl-1(3*H*)-isobenzofuranone		Inactive	
**38**	5-hydroxy-7-methoxy-4-(methoxymethyl)-6-methylisobenzofuran-1(3*H*)-one		Inactive	
**39**	5-hydroxy-7-methoxy-4,6-dimethylphthalide		Inactive	
**40**	7-methoxy-3,4,5,6-tetramethylphthalide		Inactive	
**41**	Sclerin		Inactive	
**42**	6-hydroxy-2-methyl-4-chromanone		Inactive	
**43**	Sclerotinin A		Inactive	
**44**	1-(2,5-dihydroxyphenyl)-2-buten-1-one		Antifungal activity against *M. gypseum* SH-MU-4, antiplasmodial activity against *P. falciparum*, and radical scavenging potency	
**45**	Microsphaerins A	*Microsphaeropsis* sp. F2076	Antibacterial activity against MR31SA	[16]
**46**	Microsphaerins B		-	
**47**	Microsphaerins C	*Microsphaeropsis* sp. F2078	-	
**48**	Microsphaerins D		-	
**49**	Butyrolactone I	*Microsphaeropsis olivacea*	Inactive	[17]
**50**	Graphislactone A		Activity against acetylcholinesterase (AChE)	
**51**	Ulocladol		Inactive	
**52**	Botrallin		Activity against AChE	
**53**	2,5-diacetylphenol		Inactive	
**54**	7-hydroxy-2,4-dimethyl-3(2*H*)-benzofuranone		Inactive	
**55**	Enalin		Inactive	
**56**	Palmarumycin M_1_	*Microsphaeropsis* sp. 7291	Antibacterial activity against *Bacillus megaterium*, and antialgal activity against *Chlorella fusca*	[18]
**57**	Decaspirone		Antibacterial activity against *B. megaterium* and *Microbotryum violaceum*, and antialgal activity against *C. fusca*	
**58**	Palmarumycin M_2_		Antibacterial activity against *B. megaterium*	
**59**	Papyracillic acid A		Inactive	
**60**	Papyracillic acid B		Inactive	
**61**	Papyracillic acid C		Inactive	
**62**	Microsphaeropsins B		Inactive	
**63**	Massarigenin A	*Microsphaeropsis* sp.	Inactive	[19]
**64**	Microsphaeropsones A	*Microsphaeropsis* sp. 8875	Antibacterial activity against *Escherichia coli* and *B. megaterium*, and antialgae activity against *C. fusca*	[20]
**65**	Microsphaeropsones B		Antibacterial activity and antialgae activity against *C. fusca*	
**66**	Microsphaeropsones C		Antibacterial activity against *E. coli* and *B. megaterium*, and antialgae activity against *C. fusca*	
**67**	Citreorosein		Antibacterial activity against *E. coli*, *B. megaterium*, and *M. violaceum*, and antialgae activity against *C. fusca*	
**68**	Emodin		Antibacterial activity and antialgae activity against *C. fusca*	
**69**	Fusidienol A	*Microsphaeropsis* sp. 7177	Antibacterial activity against *E. coli*, *B. megaterium*, and *M. violaceum*, and antialgae activity against *C. fusca*	
**70**	3,4-dihydrofusidienol A		Antibacterial activity and antialgae activity against *C. fusca*	
**71**	8-hydroxy-6-methyl-9-oxo-9*H*-xanthene-1-carboxylic acid methyl ester		Antibacterial activity against *E. coli* and *B. megaterium*, and antialgae activity against *C. fusca*	
**72**	Methyl 3,8-dihydroxy-6-methyl-9-oxo-9*H*-xanth- ene-1-carboxylate		Antibacterial activity and antialgae activity against *C. fusca*	
**73**	Microxanthone		Antibacterial activity and antialgae activity against *C. fusca*	
**74**	Microketides A	*Microsphaeropsis* sp. RA10-14	Antibacterial activity against *pseudomonas aeruginosa*, *Nocardia brasiliensis*, *B. anthraci*, and *Kocuria rhizophila*, and antiphytoplankton activity	[21]
**75**	Microketides B		-	
**76**	10-Norparvulenone	*Microsphaeropsis* sp. F0-5050	Anti-influenza virus against A/PR/8/34	[22]
**77**	Microsphaeropsisin	*Microsphaeropsis* sp. H5-50	Antifungal activity against *E. repens* and *U. violacea*	[9]
**78**	Microsphaeropsins A	*Microsphaeropsis* sp. 7291	Antialgal activity against *C. fusca*	[18]
**79**	Arundinols A	*M. arundinis* E12-2112	Inactive	[14]
**80**	Arundinols B		Inactive	
**81**	Arundinols C		Inactive	
**82**	1*β*-hydroxy-*α*-cyperone		Antibacterial activity against *Staphylococcus aureus*	
**83**	Betaenone derivative	*Microsphaeropsis* sp. KMPB W-22	Inhibited protein kinases activity (PKC, CDK 4, and EGF-R)	[11]
**84**	Modiolide D	*M. arundinis*	Inactive	[23]
**85**	Modiolide E		Inactive	
**86**	Modiolide A		Inactive	
**87**	Macrosphelide A	*Microsphaeropsis* sp. FO-5050	Cytotoxicity activity against HL-60	[24]
**88**	Macrosphelide B		-	
**89**	Macrosphelide C		-	[25]
**90**	Macrosphelide D		-	
**91**	Macrosphelide J		-	[10]
**92**	Macrosphelide K		-	
**93**	Modiolide	*M. arundinis* PSU-G18	Inactive	[15]
**94**	Chrysogeside D	*M. arundinis* PH 30472	Antifungal activity against *A. tenuissima*	[7]
**95**	L-755, 807	*Microsphaeropsis* sp. MF6057	Cell adhesion inhibition activity	[26]
**96**	Microsphaerones A	*Microsphaeropsis* sp. KMPB W-22	Insecticidal activity against *Spodoptera littoralis* and *Artemia salina*	[27]
**97**	Microsphaerones B		-	
**98**	*N*-2”-hydroxy-3’*E*octedecenoyl-1-*O*-β-D-glucopy-ranosyl-9-methyl-4*E*,8*E*-sphingadiene	*M. olivacea* F010	Cytotoxic activity against L1210	[28]
**99**	TAN-1496 A	*Microsphaeropsis* sp. FL-16144	Cytotoxicity activity against P815 murine mastcytoma and A59 human lung carcinoma	[29]
**100**	TAN-1496 B		-	
**101**	TAN-1496 C		-	
**102**	TAN-1496 D		-	
**103**	TAN-1496 E		-	
**104**	Betaenone derivative	*Microsphaeropsis* sp. KMPB W-22	Inactive	[11]
**105**	Fumiquinazolines L	*Microsphaeropsis* sp. CF09-11	Inactive	[13]
**106**	Fumiquinazolines N		Inactive	
**107**	Notoamide D		Inactive	
**108**	10-methyl-9*Z*-octadecenoic acid	*Microsphaeropsis olivacea*	Inactive	[30]
**109**	Glyceride		Inactive	
**110**	Microsphaerodiolin	*M. arundinis* PSU-G18	Inactive	[15]
**111**	Modiolin		Inactive	
**112**	Microsphaerol	*Microsphaeropsis* sp. 7820	Antibacterial activity against *E. coli* and *B. megaterium*	[31]

Note: “-”means the same as above.

## Data Availability

Not applicable.
